# Correction to “Association between flaunting behaviors on social media and among the general population in Bangladesh: A cross‐sectional study”

**DOI:** 10.1002/hsr2.2001

**Published:** 2024-03-19

1

Nazmunnahar, Nasim R, Mosharrafa RA, et al. Association between flaunting behaviours on social media and among the general population in Bangladesh: a cross‐sectional study. *Health Sci Rep*. 2023;6:e1701. doi:10.1002/hsr2.1701

The title will be corrected as “Association between flaunting behaviors on social media and mental health status among the general population in Bangladesh: A cross‐sectional study.”

We apologize for this error.

